# How to Improve the Survival of Transplanted Mesenchymal Stem Cell in Ischemic Heart?

**DOI:** 10.1155/2016/9682757

**Published:** 2015-11-22

**Authors:** Liangpeng Li, Xiongwen Chen, Wei Eric Wang, Chunyu Zeng

**Affiliations:** ^1^Department of Cardiology, Daping Hospital, Third Military Medical University, 10 Changjiangzhilu Road, Chongqing 400042, China; ^2^Cardiovascular Research Center, Temple University School of Medicine, Philadelphia, PA 14190, USA

## Abstract

Mesenchymal stem cell (MSC) is an intensely studied stem cell type applied for cardiac repair. For decades, the preclinical researches on animal model and clinical trials have suggested that MSC transplantation exerts therapeutic effect on ischemic heart disease. However, there remain major limitations to be overcome, one of which is the very low survival rate after transplantation in heart tissue. Various strategies have been tried to improve the MSC survival, and many of them showed promising results. In this review, we analyzed the studies in recent years to summarize the methods, effects, and mechanisms of the new strategies to address this question.

## 1. Introduction

Ischemic heart disease is the leading cause of death worldwide. Severe ischemic heart disease, especially myocardial infarction (MI) and heart failure, causes a significant loss of functional cardiomyocytes [1]. However, heart is an organ with very limited self-renewal capacity because adult cardiomyocytes can hardly regenerate [2]. Over the past decades, there has been tremendous enthusiasm in an attempt to repair cardiac tissue with stem cell transplantation [3]. Mesenchymal stem cell (MSC), with advantages in immunologic privilege, easy to be acquired, and multilineage potential, has been widely studied both in animal model and in clinical trials [4]. Low survival rate after transplantation is one of the crucial reasons accounting for the hampered cardiac repair effect of MSC. The harsh microenvironment with ischemia, inflammation, oxidative stress, and mechanical stress contributes to the great cell loss. Hence, a number of strategies have been used in attempt to overcome this obstacle. In this review, we summarize the advance of these strategies recently reported.

## 2. Characterization of MSC

MSCs are generally described as nonhematopoietic subpopulation of cells with multilineage potential to differentiate into various tissues of mesodermal origin [5]. MSCs were first identified and isolated from bone marrow (BM) more than 40 years ago [6]. They can also be isolated from other sources, such as adipose [7], synovial tissue [8], lung [9], umbilical cord blood [10], peripheral blood [11], and olfactory bulbs [12], or even in virtually all postnatal organs and tissues [13]. Among these, the most frequently used MSCs in studies for cardiac repair are BM-derived MSC (BM-MSC) and adipose-derived MSC (ADSC).

MSC has been proven to differentiate into osteoblasts, chondrocytes, and adipocytes [14]. It is also reported that MSC can transdifferentiate into mesodermal derived cell types including cardiomyocyte [15, 16], but the cardiogenic potential of MSCs is still controversial [17, 18].

MSCs are fairly heterogeneous cell population but lacks a specific marker to define MSCs [19]. According to minimum criteria that were proposed by The International Society for Cell Therapy in 2006, MSCs are characterized as (1) adherence to plastic in standard culture conditions; (2) expressing surface molecules CD73, CD90, and CD105, but in the absence of f CD34, CD45, HLA-DR, CD14 or CD11b, CD79a, or CD19; (3) a capacity for differentiation to osteoblasts, adipocytes, and chondroblasts* in vitro* [20]. Besides, MSCs possess species-specific characteristics [21], and the characteristics of MSCs may also vary according to the source of tissue [22]. For example, ADSCs were superior to BMSC with respect to maintenance of proliferating ability [23].

## 3. MSC Transplantation for Treating Ischemic Heart Disease

The first study exploring the cardiac regenerative effect of MSC was carried out in 1999 on a rat MI model induced by cryoinjury [24]. The autologous MSC was induced into cardiogenic cells by 5-azacytidine* in vitro* and transplanted into the scar of the injured hearts. The transplantation improved cardiac function, prevented remodeling, and promoted angiogenesis. In the following decades, MSCs were transplanted for treating chronic or acute ischemic heart injury in rodent models and large animals. The underlying mechanisms for the therapeutic effect include directly transdifferentiation into functional cardiomyocyte/endothelial cell, secretion of a broad spectrum of cytokine in a paracrine manner, and stimulating local cardiac stem cell proliferation [25]. It was reported that MSC can differentiate into cardiomyocyte phenotype induced by 5-azacytidine [26], coculture [15], and* in vivo* [16] models. Some observed that MSCs transdifferentiate into cardiomyocyte* in vivo*, but the cardiogenic potential of MSCs remains highly controversial. Fazel et al. injected MSCs from *β*-galactosidase (*β*-gal) transgenetic mice into the injured ischemic myocardium. As a result, there was no *β*-gal positive cardiomyocyte observed 28 days after transplantation in recipient hearts, indicating that the transdifferentiate ability of MSCs is lacking [27]. Noiseux et al. found a very few MSC-derived cardiomyocytes after transplantation, but nearly all of which were demonstrated to result from cell fusion [18]. Thus it seems that paracrine function but rather cardiogenic transdifferentiation predominantly accounts for the therapeutic effect of MSC transplantation [28].

Paracrine function of MSCs results from the MSC secretion of antiapoptotic, proangiopoietic factors (growth factors, cytokines, surface molecules, mRNA, miRNA, and exosomes) [29]. Several growth factors which consisted in conditioned medium, such as VEGF [30], FGF [31], IGF, and HGF [32], also showed cardiac regenerative capability when applied to MI model. Exosomes (or microvesicle) secretion by transplanted MSCs was reported by increasing studies [33]. Exosomes are cholesterol-rich, phospholipid vesicles of 30–100 nm enriched with microRNAs (miRNAs). MSCs-derived miRNAs-bearing exosomes are readily internalized into cardiomyocyte or and endothelial cell, resulting in cardioprotective effect via angiogenetic, antiapoptotic, or anti-inflammatory effect. MSC exosomes transferring miR-22 (can target methyl CpG binding protein 2) [34] and miR-221 (can inhibit p53-upregulated modulator of apoptosis) [35] reduce cardiomyocyte apoptosis. MSC exosomes can also reduce neutrophil and macrophage infiltration after myocardium ischemic/reperfusion injury [36].

Mitochondria transferring between MSCs and neighboring somatic cells via a “tunneling nanotube” (TNT), composed of partial membrane fusion and F-actin, was reported. This process rescued aerobic respiration of cells harboring mitochondria damage [37]. MSC also showed capacity to convey functional mitochondria to connected cardiomyocyte via TNT [38]. In a human MSCs-mouse adult cardiomyocyte coculture system, heterogeneous partial cell fusion by “tunneling nanotube” junction formation has been observed. These partial fused cells exhibited a progenitor cell-like phenotype or were described as “reprogramming/dedifferentiation.” The mitochondria conveyed through TNT were necessary for the transient partial fusion-dependent cardiomyocyte reprogramming [38]. Although the* in vivo *evidence of TNT formation and mitochondria transfer between MSC and cardiomyocyte were lacking, these findings suggested an alternative mechanism for MSC mediated beneficial effect.

Despite the underlying mechanism which remains to be clarified, the established cardioprotective effect of MSC therapy has been confirmed by most preclinical studies. In 2001, the first clinical trial of BMSCs transplantation on MI patient was conducted [39]. Thereafter, a large number of phase I/II clinical trials were designed to test the safety, feasibility, and efficiency of MSC therapy [40]. A phase II/III trial with 80 patients enrolled was conducted recently [41]. Overall, the safety and feasibility profiles of MSC therapy have been well established by most of the trials followed from 1 month to 2 years, such as POSEIDON [42], C-CURE [43], TAC-HFT [44], and MSC-HF [45]. The efficacy is also suggested, as observed improvement in 6 min walk (POSEIDON, C-CURE, and TAC-HFT), EF (C-CURE, MSC-HF), Minnesota Living with Heart Failure Questionnaire (MLHFQ) (POSEIDON, TAC-HFT), event-free survival in a 2-year follow-up (C-CURE), and reduced LV chamber (POSEIDON, C-CURE, and MSC-HF), and scar size (POSEIDON). Moreover, POSEIDON proved that allogenic MSC is comparable to autologous MSC without significant alloimmune reactions [42], supporting a favorable feasibility for MSC therapy; in addition to improved EF, a reduction in ventricular arrhythmias and improved pulmonary function were also reported in the trial employing MSC to treat patients with acute MI [46]. However, to confirm the efficacy of MSC therapy, especially the long-term outcome in patient, more rigorously designed, multicenter, long-term follow-up, well-interpreted trials with larger sample size are required. Although most of these trials have demonstrated that MSC therapy in clinical trials appears to be safe and effective [47], there are also reported investigations without observed benefits of MSC application [41]. According to the systematic review reported by Lunde et al., the MSC therapy had moderate beneficial effect on improving cardiac function (LVEF increased by 2.99% on average [48]) and limited effects on long-term effect or global end point [49].

## 4. Poor MSC Survival and Its Mechanisms

### 4.1. MSC Survival in Animal Model of MI

Among the factors which hurdle the therapeutic effect of MSC treatment, the poor survival after cell transplantation is a crucial one. Positron emission tomography (PET) tracking of MSC delivered by catheter-based transendocardial injection showed retention of approximately 6% of injected cells in porcine ischemic myocardium at 10 days after injection [50]. Toma et al. reported that less than 0.44% of MSCs survived by day 4 after engraftment in immunodeficient mouse hearts [16]. Accordingly, approximate 1% of MSCs were detected 24 hours after transplantation in rat heart with experimental MI [51].

### 4.2. MSC Survival in Ischemic Human Heart

Clinical trials have consistently demonstrated that the retention and survival of stem cells are quite low after transplantation into infarcted heart. In a small group of STEMI patients, intracoronary infusion of BMSCs labeled with 18F-FDG showed minimal retention in the infarct region (1.3% to 2.6%) when imaged by PET at 50–75 minutes after cell injection [52]. Intracoronary infusion of cultured peripheral mononuclear cells labeled with ^111^Inoxine in patients with recent STEMI resulted in activity of 6.3 ± 2.9% in the heart 24 h after injection, but it declined to 2.1% when measured 2 days later [54].

Considering that the acute MI can cause over one billion cardiomyocyte losses, one cannot realistically expect a clinically meaningful benefit from such a tiny number of residual donor cells [55]. This may account for the modest improvement of cardiac function reported in clinical trials.

### 4.3. Mechanisms for the Poor Survival of Transplanted MSC

The loss of cells number occurs in several ways: (1) a mechanical leakage of cells immediately after injection was due to the continuous compressive mechanical stress; (2) cell death, including both necrosis and apoptosis, was subsequently worsened by a harsh microenvironment of hypoxia, inflammation, and oxidative stress comprising superoxide anions and hydrogen peroxide [56]; (3) gradual loss is also attributed to the limited self-renewing rate of stem cell in ischemic myocardium, due to the lack of oxygen, inadequate nutrients, and the disrupted extracellular cell matrix (ECM).

## 5. Strategies to Improve MSC Survival

Several strategies have been explored to augment the longevity of engrafted cells in the hostile ischemic environment. The strategies are (1) more effective ways of delivery; (2) tissue engineering strategies involving scaffolds made of natural or synthetic polymers; (3) preconditioning of MSC before transplantation; (4) genetic manipulation of MSCs; (5) combined administration of MSC with another cell type or medicine.

### 5.1. Delivery Route

Stem cell can be delivered to myocardium through different ways, including peripheral intravenous infusion, direct surgical injection during open heart surgery, catheter-based intracoronary infusion, retrograde coronary venous infusion, and transendocardial injection [57, 58]. Using *γ*-emission counting of harvested organs 1 hour after cell delivery, it has been demonstrated that intramyocardial injection had the highest retention rate of delivered BMSCs. Significantly more cells were retained after intramyocardial injection (11 ± 3%) compared with intracoronary (2.6 ± 0.3%) and interstitial retrograde coronary venous infusion (3.2 ± 1%). Intramyocardial injection is the most frequently reported route for MSC therapy in animal studies, but most of the clinical trials applied catheter-based intracoronary infusion.

However, there are still some disadvantages along with needle-injection: (1) a washout of cells through channel leakage and the vascular system; (2) an inhomogeneous distribution of cells [59]. To overcome these obstacles, a cell sheet/patch based delivery method has been developed. Confluent, intact cell layers (usually 2 to 3 layers) with abundant ECM and cell-cell interaction can be acquired by culturing MSCs in thermosensitive dishes or fibrin-coated culture plate (confluent cell sheet detached spontaneously at room temperature within 30 minutes), several weeks after MI. The cell sheet was deposited onto the infarcted myocardium, and the MSC can be engrafted into myocardium and the sheet was absorbed gradually [55]. In rat model of MI, two months after the implantation, the three-layer ADSC sheet showed superior effect of cell retention compared with isolated ASDC delivered by intramyocardial injection [60].

### 5.2. Biomaterials

Due to the disrupted ECM and compressive mechanical stress, the infarcted myocardium is not an environment conducive to cell survival. Therefore, cardiac tissue engineering emerged as a promising strategy, and three-dimensional polymeric scaffolds for stem cells were developed. Scaffolds temporarily provide the biomechanical support for cells until they are able to produce their own extracellular matrix [61–63]. Scaffolds seeded with MSC showed better performance in cardiac repair than injection of MSC alone [64]. There are mainly two types of scaffold.

#### 5.2.1. Thermosensitive Hydrogel

Hydrogel as a biocompatible material was used to prevent the first wave loss of transplanted MSC due to the myocardium contraction. Hydrogels are in situ formation, biodegradable, and cell adhesive. Once delivered together with MSC, it can self-cross-link to form semigrid scaffold which could ameliorate the cell loss.

There are various types of hydrogels applied in MSC therapy for MI: (1) natural hydrogels, such as fibrin glue [65], collagen [66], alginate [67], and cardiogel [68]; cardiogel is a cardiac fibroblast-derived ECM, which was designed to mimic the natural environment suitable for transplanted MSC [68]; (2) synthetic hydrogel, including silanized hydroxypropyl methylcellulose (Si-HPMC) [69] and poly(lactide-co-epsilon-caprolactone) [64]; (3) combination of different materials in a certain ratio, such as poly(N-isopropylacrylamide) (PNIPAAm) plus single-wall carbon nanotubes (SWCNTs) [70], alginate/chitosan [71], poly(glycerol sebacate) combined with collagen [72], and hydrophobic poly(*ε*-caprolactone)-2-hydroxylethyl methacrylate (PCL-HEMA) plus PNIPAAm [73]. Hydrogel can also serve as a medium to support the diffusion of molecules [74]. Since interleukin-10 (IL-10) is an anti-inflammation cytokine, a combination of MSC, Matrigel, and IL-10 plasmids was designed to improve cell survival [75].

Hydrogel is effective in improving cell survival in stem cell therapy. In a rat MI model, intramyocardial injection of MSCs with Si-HPMC (one of the synthetic hydrogels) showed better performance in cell retention and cardiac function preservation than MSCs injection alone [76]. In a swine MI model, retention of MSC suspended in 2% alginate (a natural hydrogel) before transplantation was approximately 4-fold compared to that in control MSCs at two weeks after delivery [77]. Similarly, coinjection with fibrin glue increased ADSC survival by about 30% on a rat MI model [78].

The first clinical trial using injectable bioabsorbable scaffold (IK-5001), a solution of 1% sodium alginate plus 0.3% calcium gluconate, combined with MSCs by intracoronary delivery has been carried out (http://www.clinicaltrials.gov.: NCT01226563). This first-in-man pilot study also showed that intracoronary deployment of an IK-5001 scaffold is feasible, effective, and well tolerated in patients with STEMI [79].

#### 5.2.2. Patch/Cell Sheet

To avoid the shortcomings of needle injection, biocompatible patches seeded with MSC emerged as an alternative strategy to circumvent the lack of cell engraftment. Solid form of biomaterials (such as collagen) seeded with cells was sutured onto the surface of infarcted area. The patch can be absorbed gradually while the stem cells engrafted into the myocardium.

ADSC-cellularized sheets were implanted onto the epicardium of on chronic rat MI model [80]. No cell was detected in ADSC alone group, but cell sheet exhibited 25.3 ± 7.0% and 6.4 ± 4% engraftment rate at 1 week and 1 month after MI [60, 80]. The same group performed a head-to-head comparison of cell engraftment between the conventional injection, deposition of the bilayer myoblast cell sheet, and deposition of the myoblast cells seeded in collagen sponge in rat MI model. Both cell constructs are superior to conventional needle injection. The myoblast-seeded collagen sponge group produced the best outcome with regard to engraftment cells number and reduced fibrosis [55].

### 5.3. Hypoxic, Hyperoxic, and Pharmacological Preconditioning of MSCs

Although severe hypoxia can lead to cell death, repeated episodes of short period exposure to hypoxia (hypoxia-preconditioning) have shown conferring cytoprotective benefits [81]. Usually, MSCs were cultured under hypoxia (0.5% oxygen) or normoxic conditions for 24 hours: hypoxia-preconditioning reduced about 25% of cell death at day 1 and 40% of cell death at day 3 after delivery compared with normoxic control [82]. This effect is associated with the increased expression of prosurvival and proangiogenic factors including hypoxia-inducible factor 1 (HIF-1*α*), angiopoietin-1, vascular endothelial growth factor (VEGF), erythropoietin, Bcl-2, and Bcl-xL [82]. Moreover, hypoxia-preconditioning induced autophagy protected MSC from apoptosis, which may be also accounted for the improvement of MSC survival [83].

On the other hand, preconditioning with hyperoxia (100% oxygen) or/and Z-VAD-FMK pan-caspase inhibitor promoted MSCs viability and proliferation, by decreasing caspases 1, 3, 6, 7, and 9 expression and increasing survival genes such as Akt [84].

Sevoflurane, an inhaled anesthetic widely used in clinical anesthesia, has similar effect of hypoxia-preconditioning. Sevoflurane pretreatment minimized MSC apoptosis and the loss of its mitochondrial membrane potential induced by hypoxia, which may be mediated by HIF and Akt pathways [84].

Study also revealed that MSCs for transplantation could be preconditioned by coculturing with cells. MSC preconditioned with cardiomyocytes in culture exerted enhanced therapeutic effect compared with MSC alone [85]. The hetero-cell-to-cell connection altered the MSC paracrine of cardioprotective soluble factors such as VEGF, HGF, SDF-1*α*, and MCP-3.

Preconditioning of MSCs with TGF-*α* enhanced the VEGF secretion of transplanted MSC* in vivo*, thereby enhancing MSCs' ability to protect myocardium from IR injury [86]. Platelet-derived growth factor-BB (PDGF) treatment of MSCs resulted in rapid activation of both Akt and ERK and upregulated VEGF. Thus, MSCs with PDGF preconditioning exhibited a greater capacity of functional recovery compared with naïve MSCs in I/R injured heart [87].

Preconditioning can be operated* in vitro* prior to transplantation, which circumvents the side effect caused by other approaches such as genetic manipulation. Since the forced gene manipulation in stem cells raises concern about the safety in long-term effect, the continuous overexpression of gene in MSCs may be harmful if the microenvironment switches to different stage [88].

### 5.4. Genetic Modification of MSCs

Genes related to cardiac protection from I/R injuries such as Akt and Integrin-linked kinase [89] and genes involved in apoptosis such as Bcl-2 [90] promoted stem cell survival in ischemic myocardium.

Akt has been well documented among genetic approaches. In both rat and porcine model of MI, transplantation of Akt-engineered MSCs led to improved LVEF and reduced scar size and fibrosis; this is because not only were Akt-engineered MSCs more resistant to apoptosis [18, 91], Akt modification also enhanced MSC secretion of paracrine factors such as VEGF, IGF-1, and FGF-2 [92]. A double overexpression system in MSCs comprising Akt and angiopoietin-1 (an important modulator in angiogenesis) further improved cell survival [93]. Overexpression of heat shock protein 20 (Hsp-20) [94], secreted frizzled related protein 2 (sFRP2), a modulator of the Wnt signaling [95], survivin [96], heme oxygenase (HO-1) [97], GSK-3*β* [98], ERBB4 [99], CCR-1 [100], and serum derived factor-1 (SDF-1) [101] in MSC had similar beneficial effect on cell survival.

We have demonstrated previously that silencing of prolyl hydroxylase domain protein 2 (PHD2) enhanced ADSC survival after transplantation into murine ischemic myocardium, by maintenance of active HIF-1*α* [102]. PHD2 silencing can also enhance ADSC paracrine antiapoptotic effect on cardiomyocytes against ischemia via NF-*κ*B signaling. Similarly, GATA-4 overexpression in MSCs increased both MSC survival and angiogenic potential in ischemic myocardium [103].

In addition to coding gene, microRNA (miR) has been explored for stem cell therapy with its multitargets property. Overexpression of miR-1 in MSCs promoted their survival 2-3-fold at 7 days after transplantation, leading to more conducive repair of infarct injury and improved heart function. miR-1 promoted MSC survival via regulating caspase 9, Bcl-2, and Bax [104]. miR-210 engineering has similar effect in MSCs through antioxidative c-Met pathway [105].

### 5.5. Cotransplantation of MSCs with Other Cells

CS/PCs, such as c-kit positive residential progenitor cells in heart [106], emerged as another potential cell source for cardiac repair [106]. Clinical trial of CS/PCs reported encouraging results in preserving cardiac function for patients with ischemic cardiomyopathy [107]. Based on the observation that MSC can stimulate endogenous CS/PCs proliferation [25] and regulate CS/PCs niches [108], studies have been performed to explore the effect of using MSCs together with CS/PCs [109, 110]. In a porcine model of MI, a combination of human CS/PCs and MSCs labeled with iron oxide for CMR imaging was delivered into myocardium 14 days after MI. CMR revealed that this combination was 7-fold greater cell engraftment than either cell type alone, thereby further reduced scar size, and improved cardiac function [110].

Inflammatory status in acute stage is another important factor causing the low retention of transplanted MSCs. Previous studies have shown that CD4^+^CD25^hi^FoxP3^+^ T regulatory (Treg) cells have a potential to suppress inflammation, thus providing a favorable environment for MSC engraftment [53, 111]. The cotransplantation of autologous Treg cells with MSC dramatically increased the MSC survival rate and proliferation in a porcine MI model with no deleterious side effects observed [112].

### 5.6. Administration of MSCs with Medication

Statins have some cardioprotective function independent of their lipid-lowering ability. They can protect endothelial function, increase nitric oxide bioavailability, and exert antioxidant/anti-inflammatory effects [113–117]. Combination of Simvastatin (0.25 mg/kg/d) and MSC transplantation (3 × 10^7^ cells per animal) showed approximately 4-fold higher MSC survival rate compared with MSC alone. This was due to the fact that oxidative stress and inflammatory response were significantly reduced in the infarcted regions by Simvastatin [118]. Other groups reported that Rosuvastatin improved survival of ADSCs after transplantation into infarcted hearts. Improved cardiac function and reduced fibrosis were observed in Rosuvastatin plus ADSC group. Bioluminescence imaging and histological staining* in vivo* revealed that Rosuvastatin (20 mg/kg per day for 28 days) enhanced the survival of engrafted ADSC approximately 1.3-fold compared to MSC alone. This was associated with the idea that Rosuvastatin increased Akt, ERK phosphorylation, promoted the subsequent FoxO3a phosphorylation and nuclear export, and decreased the proapoptotic proteins in ADSCs [119].

### 5.7. Another Optimization for MSC Therapy

#### 5.7.1. Time Point

Li and colleagues compared the time points for optimizing MSC efficacy. Among 1 h, 1 week, and 2 weeks delivery after acute MI, time point of 1 week exhibited the most abundant MSC survival. Delivery at a later time point may lead to impeded cell retention, whereas cell administration too early may lead to poor engraftment due to the intensive inflammatory response in the acute stage [120]. According to an analysis of 7 randomized controlled trials with 660 patients with MI undergoing emergent percutaneous coronary intervention and receiving intracoronary BMSC transplantation, the effect of BMSC delivery at 4 to 7 days was superior to that within 24 hours [121].

#### 5.7.2. Intactness

Adhesion is important for cell survival. Disruption of cell-ECM contact by trypsinization prior to cell transplantation may impair cell viability and facilitate apoptosis. Recently, improving MSC survival after transplantation with effective adhesion attracts much attention. MSCs were expanded on microcarrier beads in spin culture and directly transplanted, which avoided trypsinization and detachment of cell-ECM interaction, showing significantly less apoptosis than trypsinized control cells [122].

#### 5.7.3. Extracorporeal Shock Wave

Genetically overexpression of SDF-1 in MSC can improve cell retention in ischemic myocardium [123]. Extracorporeal shock wave, similar to that used to treat nephrolithiasis, has been experimentally demonstrated to increase homing factor such as SDF-1 in target tissue [124]. Whether shock wave treatment can increase MSC retention requires further investigation. However, the phase I/II, double-blind, randomized, placebo-controlled trial CELLWAVE (NCT NCT00326989) conducted among patients reported that patients with chronic heart failure receiving shock wave treatment prior to intracoronary BM-MNC infusion had a modest but significant improvement in LVEF compared to shock wave/placebo infusion [125]. Although the cell retention has not been evaluated in this trial, extracorporeal shock wave remains a strategy of interest in future.

## 6. Discussion

**Figure 1:**
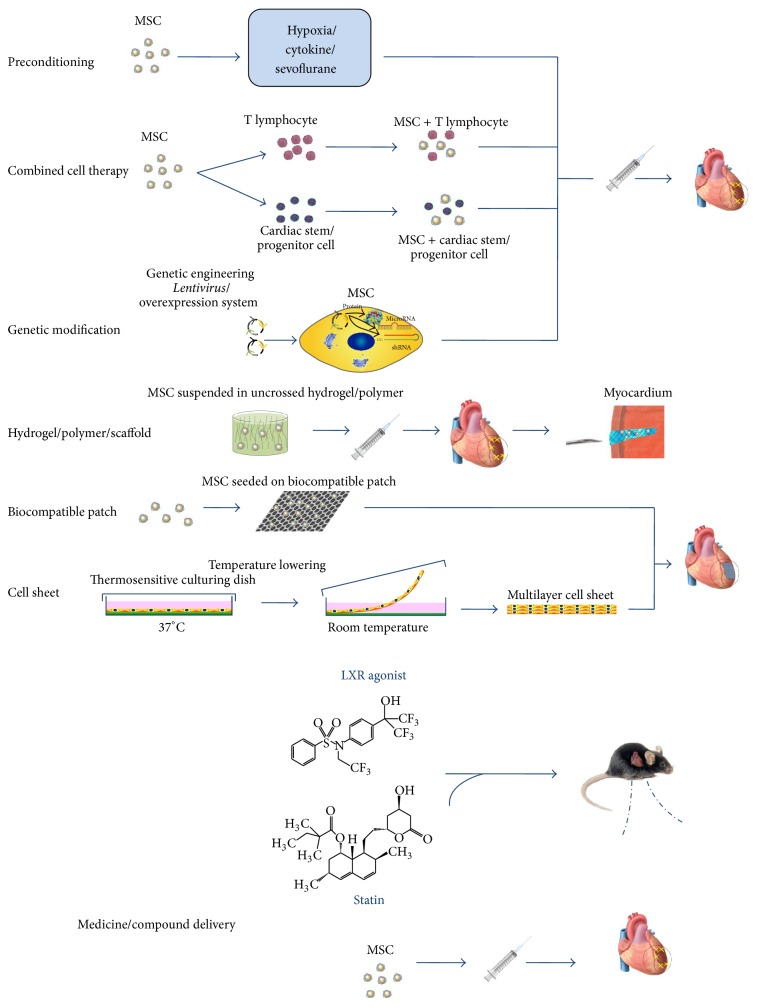
Schematic cartoon to illustrate the strategies to improve survival of MSC in ischemic myocardium. MSC can be either pretreated by hypoxia/cytokine or genetically modified before delivery to myocardium. Hydrogel/polymer with suspended MSC forms a semigrid scaffold when injected into the myocardium, which improves cell retention. MSC can be also seeded on biocompatible patch or form cell layer by culturing in thermosensitive dish; both of them can be deposited or sutured onto the epicardium of the infarcted area. Combined with another cell type (CSC, Treg cell) or medicine/compound (statin), they improve the survival of MSC.

The poor viability of transplanted MSCs hampers their therapeutic efficacy for cardiac repair. A number of strategies have been conducted to augment the longevity of engraft cell in the hostile environment. We summarized the efficacy of promoting MSC survival with different strategies (Table 1, Figure 1). Myocardium injection has superior cell retention compared with other traditional routes such as catheter-based intracoronary infusion. MSCs seeded biocompatible materials scaffold delivered by injection or suturing to the epicardium hold promising potential. Biomaterials such as hydrogel can prevent the first wave loss of transplanted MSC due to the myocardium contraction. Preconditioning and genetic modification of MSCs can enhance the resistance of MSCs against hypoxia, oxidation, and inflammation. MSC transplantation together with CS/PC or Treg cells showed enhanced cell engraftment. Statin improves MSC survival after transplantation based on its multipotent function. A limitation of this review is that the efficacies of approaches improving cell retention are difficult to be compared with each other. This is partially due to the measurements of cell retention in different studies.

As paracrine effects are nowadays considered as predominant therapeutic effect of MSCs [126], there is an attempt to apply functional fraction of conditioned medium (or its components such as exosome) of cultured MSCs instead of direct cell delivery to treat heart disease [33–35]. However, some effects, including mitochondria transfer effect between MSC and cardiomyocytes [38], cannot be mimicked by paracrine factors alone. Further, the strategies acquired from MSC studies to promote cell survival have broader significance. Some stem cell types such as embryonic stem cell and inducible pluripotent stem cell (iPSC) have indisputable capacity to generate new cardiomyocytes, which are conducted in preclinical studies. The optimized methods for MSC transplantation can be tested in iPSC-derived cell treatment in ischemic myocardium, leading to greater efficacy for cardiac regeneration [127–134].

## Figures and Tables

**Table 1:** Strategies to improve MSC survival in ischemic myocardium.

Treatment	Species	Cell amount	Labeling and measurement	Survival						Unit	Fold changes (approximately)			References
*Genetic modification *														

Bcl-2 overexpression	Rat	6 × 10^6^	BrdU labeling and immunofluorescence	4 days		3 weeks		6 weeks		Brdu positive per 1000 nuclei	4 days	3 weeks	6 weeks	
				Bcl-2 MSC	Vector-MSC	Bcl-2 MSC	Vector-MSC	Bcl-2 MSC	Vector-MSC		2.2 fold	1.9 fold	1.2 fold	[90]
				325	100	180	57	110	50					

GATA4 overexpression	Rat	1.5 × 10^6^	SRY gene quantification	4 days						Cells per mg tissue				
				GATA4-MSC			Vector-MSC				2.8 fold			[102]
				4			1.4							

HSP20 overexpression	Rat	1 × 10^6^	SRY gene quantification	4 days						Fold				
				Hsp20-MSC			GFP-MSC				2 fold			[94]
				2			1							

Akt overexpression	Mouse	5 × 10^5^	GFP overexpression and immunofluorescence	3 days		7 days		14 days		GFP+ cells	3 days	7 days	14 days	
				Akt-MSC	GFP-MSC	Akt-MSC	GFP-MSC	Akt-MSC	GFP-MSC		>2 fold	>6 fold	>5 fold	[18]
				510	220	100	18	7	1					

Survivin overexpression	Rat	2 × 10^6^	PCR for GFP	7 days				24 days		Ratio of GFP/GAPDH	7 days		28 days	
				Survivin-MSC	GFP-MSC			Survivin-MSC	GFP-MSC		2.5 fold		4.3 fold	[96]
				0.62	0.25			0.56	0.1					

HO-1 overexpression	Mouse	1 × 10^6^	SRY gene quantification	4 hours		24 hours		1 week		Implanted cells	4 hours	24 hours	1 week	
				HO-1-MSC	LacZ-MSC	HO-1-MSC	LacZ-MSC	HO-1-MSC	LacZ-MSC		3.5 fold	1.5 fold	7.5 fold	[97]
				70%	20%	20%	8%	15%	2%					

GSK-3*β*	Mouse	1.5 × 10^5^	GFP overexpression and immunofluorescence	12 weeks						GFP^pos^cells/mm^2^				
				GSK-3*β*-GFP-MSC			GFP-MSC				—			[98]
				25.8			0%							

ERBB4 overexpression	Mouse	3 × 10^5^	GFP overexpression and immunofluorescence	4 weeks						GFP^pos^cells/mm^2^				
				ERBB4-GFP-MSC			GFP-MSC				5.6 fold			[99]
				28			5							

CCR1 overexpression	Mouse	3 × 10^5^	GFP overexpression and immunofluorescence	72 hours						Cells/mm^2^				
				CCR1-GFP-MSC			GFP-MSC				2.5 fold			[100]
				100			42							

mir-1 overexpression	Mouse	5 × 10^6^	SRY gene quantification	1 week						Cells/mg tissue				
				mir-1-MSC			MSC				2.26 fold			[103]
				3.4 × 10^3^			1.5 × 10^3^							

PHD2 silencing	Mouse	1 × 10^5^	GFP overexpression and immunofluorescence	4 weeks						% of cell delivered				
				shPHD2-MSC			MSC				5.4 fold			[102]
				0.65 ± 0.09%			0.12 ± 0.04%							

SDF-1 overexpression	Rat	5 × 10^5^	DAPI labeling	1 week						Cells/mm^2^				
				SDF-1-MSC			EGFP-MSC				4 fold			[101]
				200			55							

sFrp2 overexpression	Mouse	2.5 × 10^5^	GFP counting	30 days						Cells				
				sFrp2-MSC			GFP-MSC				4 fold			[94]
				8			2							

*Hydrogel/polymer/scaffold *														

PNIPAAm + SWCNTs + BMSC	Rat	2 × 10^6^	Dil labeling	1 week						Cell numbers/filed				
				PNIPAAm + SWCNTs + BMSC			PBS + BMSC				1.5 fold			[70]
				33			21							

Fibrin glue + ADSC	Rat	5 × 10^6^	DAPI labeling	24 hours						% of cell delivered				
				Fibrin + ADSC			ADSC				1.4 fold			
				19.10 ± 3.13%			14.16 ± 2.73%							[78]
				4 weeks (grafted size)						Grafted size				
				Fibrin + ADSC			ADSC				1.96 fold			
				11.52 ± 2.34%			5.85 ± 1.35							

Alginate + MSC	Swine	2 × 10^6^	Dil labeling	2 weeks						Area of engraftment%				
				Alginate + MSC			MSC				4 fold			[77]
				12			3							

Fibrin glue + skeletal myoblast	Rat	5 × 10^6^	Dil labeling	5 weeks						% of the infarcted area				
				Fibrin glue + skeletal myoblast			BSA + skeletal myoblast				2.2 fold			[65]
				9.7 ± 4.2%			4.3 ± 1.5%							

a-Cyclodextrin/(MPEG-PCL-MPEG)	Rabbit	2 × 10^7^	DAPI labeling	4 weeks						Cells/mm^2^				
				a-Cyclodextrin/(MPEG-PCL-MPEG) + MSC			MSC				2.5 fold			[127]
				2150 ± 235			845 ± 156							

Dex-PCL-HEMA/PNIPAAm + BMMNC	Rabbit	1 × 10^7^	DAPI labeling	48 hours						% of cell delivered				
				Dex-PCL-HEMA/PNIPAAm + BMMNC			BMMNC				1.75 fold			[73]
				21			12							

*Patch/cellsSheet *														

Collagen scaffold loaded with IL-10 plasmid	Rat	—	Dil labeling	4 weeks						Cells/mm^2^				
				Scaffold + IL-10 plasmid + MSC		Scaffold + MSC	Scaffold alone				>5 fold			[75]
				13		2.5	0							

Collagen patch seeded with ADSC	Rat	3.5 × 10^5^	GFP counting	1 week (engraftment)						% of cell delivered				
				Collagen patch seeded with ADSC			ADSC alone				—			
				25.3 ± 7.0%			0							[80]
				1 month (engraftment)						% of cell delivered				
				Collagen patch seeded with ADSC			ADSC alone				—			
				6.4 ± 4%			0							

MSC cell sheet	Rat	4 × 10^6^	SRY gene quantification	3 days			28 days			Implanted cells	3 days		28 days	
				MSC cell sheet		MSC injection	MSC cell sheet		MSC injection		>11 fold		>18 fold	[128]
				56.0 ± 7.2%		5.0 ± 1.0%	9.1 ± 1.9%		0.5 ± 0.2%					

Triple-layer autologous ADSC sheet	Rat	1 × 10^7^	GFP counting	2 months						Scoring				
				Triple-layer autologous ADSC sheet			ADSC				—			[60]
				2-3 (moderate or large number of cells)			0-1 (no or minimal amount of cells)							

Collagen sponge seeded with myoblast	Rat	5 × 10^7^	Human gene PCR	4 weeks (engraftment)						DNA ng/*μ*L (mean [min, max])				
				Collagen sponge seeded with myoblast		Myoblast cell sheet	Myoblast injection				—			[55]
				6.2 [0.00, 17.40]		0 [0.00, 88.00]	0 [0.00, 2.60]							

*Combined therapy *														

MSC + CS/PC coimplantation	Rat	2 × 10^8^/1 × 10^6^	Alu immunofluorescence	4 weeks						Cells/cm^3^				
				MSC + CS/PC		MSC alone	CPC alone				7 fold			[110]
				7.5		2	0							

Rosuvastatin + MSC	Mouse	1 × 10^6^	GFP overexpression and bioluminescence imaging	2 weeks						Photons/s/cm^2^/sr				
				Rosuvastatin + MSC			MSC alone				1.75			[119]
				0.71 ± 0.02 × 10^5^			0.40 ± 0.05 × 10^5^							

Atorvastatin + MSC	Rat	5 × 10^6^	SRY gene quantification	4 weeks						Cells/per high power field				
				Atorvastatin + MSC			MSC alone				4 fold			[129]
				22.0 ± 1.7			5.7 ± 1.2							

Simvastatin (SIMV) + MSC	Swine	3 × 10^7^	DAPI labeling	6 weeks						Cells/per 5 sections				
				SIMV + MSC			MSC				4~5 fold			[113]
				310.6 ± 83.8			70.5 ± 22.3							

LXR agonist T0901317	Mouse	1 × 10^6^	GFP overexpression and bioluminescence imaging	4 weeks						Photons/s/cm^2^/sr				
				T0901317 + ADSC			ADSC alone				5.4 fold			[130]
				0.27 ± 0.05 × 10^5^			0.05 ± 0.03 × 10^5^							

IP6Ks inhibitor TNP	Mouse	1 × 10^6^	GFP overexpression and immunofluorescence	2 weeks						Cells/mm2				
				TNP + MSC			MSC alone				2.5 fold			[131]
				366 ± 34.2			114 ± 17.8							

Ghrelin pretreatment	Mouse	7 × 10^5^	GFP overexpression and bioluminescence imaging	3 weeks						Photons/s/cm^2^/sr				
				ADSC pretreated with grhelin			ADSC				1.75 fold			[132]
				0.7 ± 0.02 × 10^5^			0.4 ± 0.03 × 10^5^							

Ad-HIF-*α* + MSC	Mouse	1 × 10^6^	SRY gene quantification/bioluminescence imaging	3 weeks						Cell survival rate				
				Ad-HIF-*α* combined with MSC			MSC alone				2.14 fold			[133]
				1.50%			0.70%							

HGF/VEGF + MSC	Mouse	0.5 × 10^6^	Bioluminescence imaging	3 days			4 days			Fold changes	3 days		4 days	
				HGF + MSC		MSC alone	VEGF + MSC		MSC alone		HGF		VEGF	[134]
				1.67 ± 0.43		1	1.44 ± 0.55		1		1.67 ± 0.43		1.44 ± 0.55	
